# *Artifact3-D*: New software for accurate, objective and efficient 3D analysis and documentation of archaeological artifacts

**DOI:** 10.1371/journal.pone.0268401

**Published:** 2022-06-16

**Authors:** Leore Grosman, Antoine Muller, Itamar Dag, Hadas Goldgeier, Ortal Harush, Gadi Herzlinger, Keren Nebenhaus, Francesco Valetta, Talia Yashuv, Nir Dick

**Affiliations:** Institute of Archaeology, Mount Scopus, The Hebrew University of Jerusalem, Jerusalem, Israel; University at Buffalo - The State University of New York, UNITED STATES

## Abstract

The study of artifacts is fundamental to archaeological research. The features of individual artifacts are recorded, analyzed, and compared within and between contextual assemblages. Here we present and make available for academic-use *Artifact3-D*, a new software package comprised of a suite of analysis and documentation procedures for archaeological artifacts. We introduce it here, alongside real archaeological case studies to demonstrate its utility. *Artifact3-D* equips its users with a range of computational functions for accurate measurements, including orthogonal distances, surface area, volume, CoM, edge angles, asymmetry, and scar attributes. Metrics and figures for each of these measurements are easily exported for the purposes of further analysis and illustration. We test these functions on a range of real archaeological case studies pertaining to tool functionality, technological organization, manufacturing traditions, knapping techniques, and knapper skill. Here we focus on lithic artifacts, but the *Artifact3-D* software can be used on any artifact type to address the needs of modern archaeology. Computational methods are increasingly becoming entwined in the excavation, documentation, analysis, database creation, and publication of archaeological research. *Artifact3-D* offers functions to address every stage of this workflow. It equips the user with the requisite toolkit for archaeological research that is accurate, objective, repeatable and efficient. This program will help archaeological research deal with the abundant material found during excavations and will open new horizons in research trajectories.

## 1. Introduction

The study of artifacts is fundamental to archaeological research. The features of individual artifacts are recorded, analyzed, and compared within and between contextual assemblages. The results of these endeavors form the basis of the higher-level interpretations and reconstructions of past human behavior, cultural evolution, and history. Hence, without precise, objective, and repeatable data acquisition methods that comprehensively record key artifact attributes and metrics, the evidence gathered from the abundant material found during excavations is problematic.

Interpreting the results of artifact assemblages subjected to various analyses, mostly in a comparative nature, regularly revolves around the identification and description of variability (or the lack thereof). In the most basic sense, variability in the attributes of artifacts over time or space is regularly interpreted as indicating technological, cultural, or functional distinction and transition, while homogeneity is interpreted as indicating continuity or influence [e.g. 1–6]. Thus, the degree and nature of variability in artifact attributes form the basis for archaeological interpretations of the past. How well we measure this variability is therefore crucial to the discipline.

Until recently, the capacity to document and express relevant, culturally indicative morphological features of artifacts in a quantitative manner and high-resolution were extremely limited. Solutions such as classification into discrete categories, namely typology, are based on qualitative, intuitive notions which are described lexically and lack formal, quantitatively measurable definitions [7–9]. The lack of continuous quantitative variables substantially reduces both the statistical power and the overall credibility of subsequent analyses performed on assemblages. In contrast, the acquisition of quantitative data was limited to simple linear distance measurements, such as length, width, and thickness. These measurements not only suffer from low accuracy with respect to their position relative to the artifact and each other, but also fail to capture the complex morphological phenomena in artifacts, regularly used in cultural reasoning and interpretation [10–12].

The exponential increase in computing capacities, coupled with the proliferation of 3D digital scanning technologies in the past few years render computational approaches as the natural candidates for addressing these problems. These offer an efficient and accurate solution to digitally record the morphological information of archaeological artifacts to make it suitable for further methods of computational display and analysis [13, 14].

At the *Computational Archaeology Laboratory* of the *Hebrew University of Jerusalem*, we have worked throughout the last decade towards technological innovations in response to relevant archaeological questions, in a team consisting of archaeologists, applied mathematicians, and computer scientists. 3D modelling technology along with computational analytical methods are harnessed to describe and analyze lithic and other artifacts. These are used to provide innovative research methodologies for obtaining new insights into questions for which standard avenues of research have reached an impasse.

We have developed methods to compare artifacts based on morphological attributes derived from their whole 3D shape at different levels of resolution. For instance, we compared earlier and later Acheulean handaxes using their intrinsic 3D properties like the center of mass and volume, that can only be reliably measured with computational methods [15]. Additionally, we used quantitatively measured the asymmetry and the roughness of handaxe outlines to develop a temporal sequence that identified the Palaeolithic site of Lake Zihor as another milestone on the migration route of the early hominids from Africa to Eurasia [16]. These metrics were similarly used to model the role of post-depositional damage on bifaces [17]. Beyond bifaces, we also developed a method for automatically segmenting, displaying, and analyzing the scars on the dorsal surfaces of stone tools [18, 19]. Most recently, we used these 3D methods to measure and illustrate Epipaleolithic stone tool assemblages from the Levant [20], as well as precisely compare the edge angles of microliths from these sites [21].

The present manuscript offers the archaeological community these, and more, developments in one software package: *Artifact3-D*. The package integrates all these functionalities into a user-friendly and intuitive graphical user interface (GUI) which does not require high proficiency in computer science. Here, we demonstrate this software’s utility by describing the application of each function to a real archaeological case study. For instance, we explore how an object’s center of mass reflects various aspects about its manufacturing technology and intended functionality. We also show how precise measurements of length, surface area, and 2D sections can be used to reconstruct reduction sequences and reduction intensity. Precise angle measurements can be used to track the manufacturing traditions of different prehistoric cultural groups. The asymmetry of Levallois flake outlines, coupled with the curvature of their ventral surfaces, is used to compare the motor control and manual skills of different experimental knappers. Lastly, the invasiveness of Levallois flaking strategies is reconstructed using a scar segmentation algorithm. These case studies serve as examples of how the *Artifact3-D* software can be used to respond to real archaeological and experimental research questions. We hope this will contribute to the promotion of objective, computational methods for documentation and analysis in archaeology and open the way to address completely new research questions in an interdisciplinary interchange with mathematics and computer science.

## 2. *Artifact3-D*

### 2.1 Background

The *Artifact3-D* software was developed at the *Computational Archaeology Laboratory* of the *Hebrew University of Jerusalem* in the Matlab IDE. Here, we make the 2022 version of the software, compatible with systems running Microsoft Windows 10 or higher operating systems, freely available for academic use. The software is installed using a simple installation wizard which automatically downloads and installs the Matlab runtime environment as part of the installation process if that is required. The software and its associated user manual (S1 File) are available for download at the following link; https://sourceforge.net/projects/artifact3-d/.

While some specific functions of this software have been discussed before [13, 15, 19, 21, 22], here we present for the first time the software in its complete and most up-to-date form, alongside case studies to demonstrate the software’s utility for resolving real archaeological research questions.

### 2.2 Importing and positioning scans

**Fig 1:**
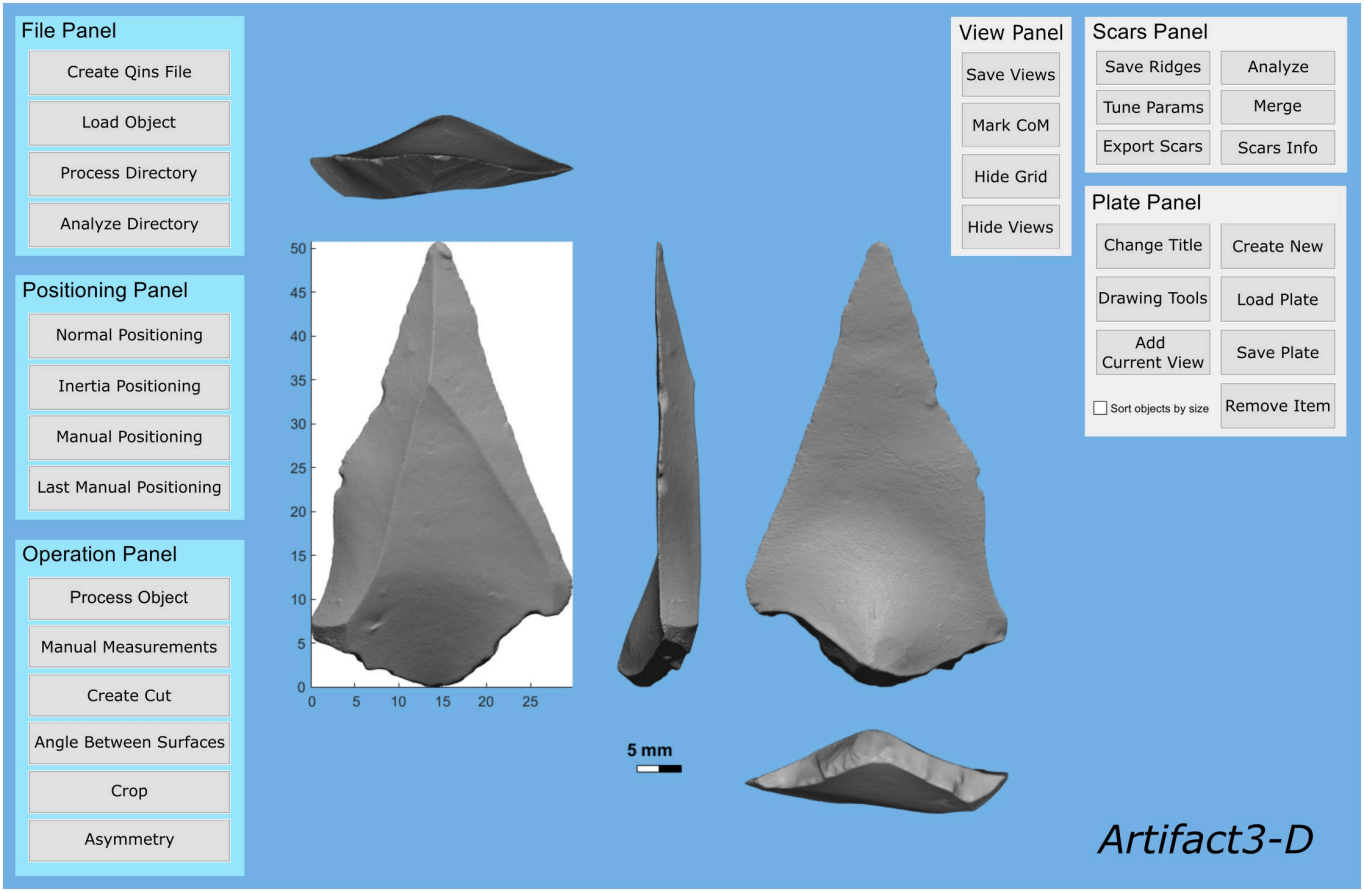
The primary interface of the *Artifact3-D* program, showing the main functions that can be conducted on an artifact.

As input, the software takes 3D scans (in .wrl, .stl, and .ply format), which can be imported to the primary user interface (Fig 1) as individual files (*File Panel—Create Qins File*) or as an entire folder of scans (*File Panel—Process Directory*). This means that entire assemblages can be automatically imported and analyzed with a single click and left running in the background. By loading a 3D model (S2 File) of an artifact into the software, whether individually or an entire directory, a Qins file (.mat) is created. This file contains the 3D coordinates of the vertices and faces of the model as well as positioning matrices that govern how the artifact is oriented in the interface. Previously imported scans can also be reloaded by selecting its Qins file (*File Panel—Load Object*), which will appear with the orientation that was previously selected.

While high-resolution scans procured from structured-light scanners are typically used in the *Computational Archaeology Laboratory*, the program has also been successfully used with lower resolution scans and even photogrammetry models. *Artifact3-D* was primarily developed with lithic artifacts in mind, but the software has also been used with a wide range of non-lithic materials, including bone tools [23–25], ground-stone tools [26], metal tools [27], paleobotanical remains [28], and several decorative and figurative items [29–31].

As a basis for all subsequent analyses, *Artifact3-D* automatically positions each artifact objectively and repeatably based on the intrinsic properties of the object. The two different available methods of automatically positioning artifacts have been described in detail before [15, 32]. The first method (*File Panel—Normal Positioning*) uses the normal vectors and the surface area of each triangle in the mesh model. These are used to calculate the surface tensor whose eigenvectors reflect three orthogonal planes minimizing asymmetry under reflection. The model is then rotated so that the eigenvectors would correspond to the X, Y, and Z axes of a common Euclidean space [32].

The second method (*File Panel—Inertia Positioning*) uses the radial vectors to each vertex of the mesh model along with the respective volume from the center of mass (assuming a uniform distribution of mass). This data is used to calculate the inertia tensor, whose eigenvectors reflect the maximal principal moment of inertia, namely the axis about which rotational acceleration requires the least torque [15]. It should be noted that given the assumption of uniform mass distribution both methods reflect to a large degree the planes of minimal asymmetry. The small, generally negligible, differences between them stem from higher sensitivity of the former to surface roughness (irregularity) and in any sense both methods are equally valid with no a priori reason to prefer one over the other.

Lastly, manually orientating the artifact (*File Panel—Manual Positioning*) is also possible for those wanting to follow traditional positioning guidelines that meet typological and/or technological requirements, rather than the intrinsic properties of the artifact. The object can be manually positioned according to any criteria chosen by the user. To do so, every orthogonal projection of the artifact can be altered in increments of 1, 2, 5, 10, 45, and 90°. If necessary, this allows the user to quantify or standardize how this manual orientation deviates from the automatic positioning.

## 3 Functions

### 3.1 Process object

**Fig 2:**
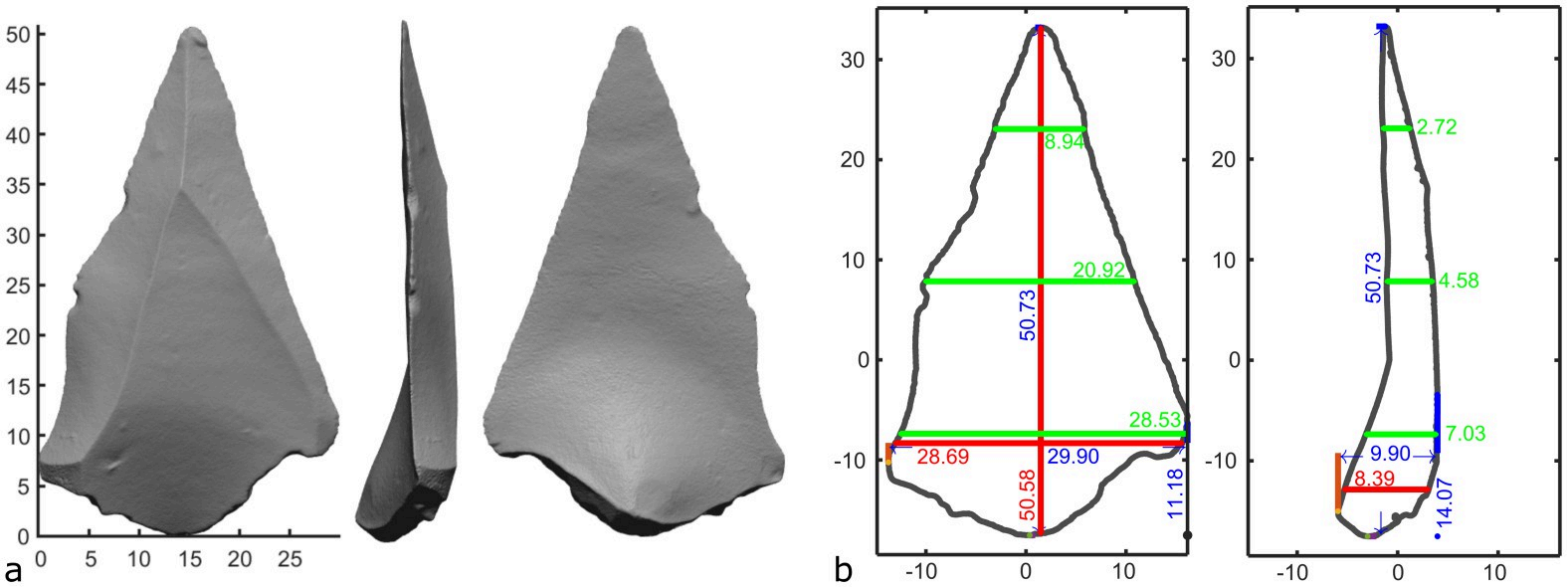
An example of the images produced during the *Process Directory* and *Process Object* function, showing the 3D scan in different orthogonal projections (a), as well as the key linear measurements (in mm) taken on the object (b).

Once an object is suitably positioned, a suite of metrics can be automatically calculated (*Operation Panel—Process Object*). These include several orthogonal measurements, the object’s volume, as well as values related to the object’s center of mass (CoM). After processing the object, the software will automatically export these values, as well as graphical representations of the object and some key measurements (Fig 2). The figures are exported in .jpg and .fig formats, while the data is exported as .xlsx and .mat files.

Based on the artifact’s automatic or manual positioning, a set of orthogonal linear measurements are produced, like those typically taken using calipers. These measurements include maximum length, width, and thickness, as well as width and thickness at one-fifth length, four-fifths length, and the midsection. The software automatically outputs a number of indices based on these measurements. Although these measurements were chosen to conform to those required for Bordes’ [7] and Roe’s [33, 34] original classification of handaxe shape, they also provide useful, precise, and objective quantifications of any object. Alongside these measurements, the volume of the object is also calculated, a metric that can only be reliably calculated using 3D scan data.

### 3.2 Centre of Mass (CoM)

The CoM of any object has significant techno-functional implications. For instance, a tool’s distribution of mass is responsible for its balance, and thus influences its optimal functionality [13]. A tool with a CoM near its use-edge tends to be suited for a striking motion. Meanwhile, tools requiring deft manipulation and dexterity tend to be heavier near their base, placing the CoM closer to the hand during use. Lastly, projectiles, like arrows, typically require a central CoM to ensure accurate trajectories.

An artifact’s CoM, automatically calculated as part of its positioning, is among the suite of metrics exported using the *Operation Panel—Process Object* function, and its position can also be displayed on the interface using the *View Panel—Mark Centre of Mass* button. This value is calculated by multiplying the surface area of each triangular face by the vector of the center of those triangles followed by the division of the total surface, and as such it represents the volumetric center of the object. For artifacts made from sufficiently homogenous raw materials, this value reliably approximates its CoM, which can have significant techno-functional implications.

In *Artifact3-D*, the object’s 3D coordinates are always centered around the CoM, meaning its xyz coordinates are always 0, 0, 0. Of particular interest is the relationship between the CoM and the center of the bounding box (CoBB). The bounding box surrounds the artifact by enclosing the artifact in a rectangular prism whose boundaries correspond to the artifact’s maximum dimensions (length, width, and thickness). The CoBB represents the center of the three orthogonal sides that form the ‘box’. Discrepancies between the CoM (0, 0, 0) and the CoBB reveal the artifact’s volumetric distribution and its deviation from perfect reflectional symmetry on each of the three perpendicular physical planes [15].

**Fig 3:**
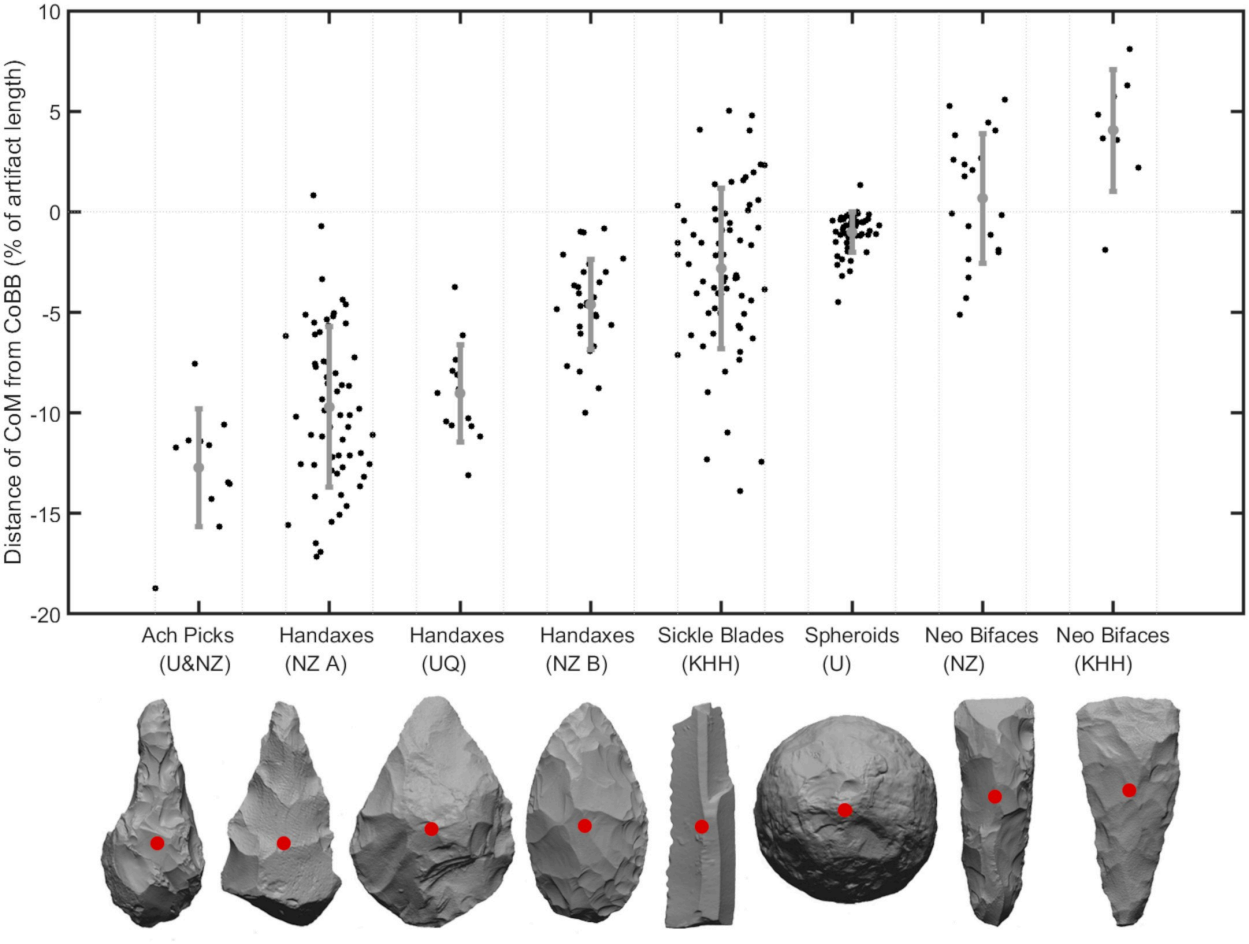
Jitter plot comparing the CoM with the CoBB for (in order) picks from Ubeidiya and Nahal Zihor, handaxes from Nahal Zihor Group A, handaxes from Um Qatafa, handaxes from Nahal Zihor Group B, sickle blades from Kfar HaHoresh, Spheroids from Ubeidiya, Neolithic bifaces from Nahal Zihor, and Neolithic bifaces from Kfar HaHoresh. The y-axis shows the difference in y-coordinates, and the x-axis shows the difference in x-coordinates, normalized by artifact length and width respectively. Grey vertical lines show the maximum variation in CoM and CoBB x-coordinates. The mean values and standard deviation error bars are shown in blue.

To demonstrate the significance of this metric, Fig 3 explores the variability of CoM values for different stone tool types. This jitter plot shows the y-axis difference between the CoM and the CoBB as a percentage of the artifacts’ range on that axis to normalize for size. The jitter of the points along the x-axis also shows the variation between the CoM and CoBB for the x-coordinates, normalized by artifact width. A number of different artifact types are shown in Fig 3, including picks, handaxes, sickle blades, spheroids, and Neolithic bifaces. These artifacts are from Ubeidiya [35, 36], Nahal Zihor [16, 37], Um Qatafa [38–41], and Kfar HaHoresh [42, 43].

The differences in CoM location among these different assemblages reveals the significance of this metric. The tendency for Acheulean bifaces to possess distally skewed CoM values compared with Neolithic axes and adzes hints at key functional differences between these tool types. While the exact functions of these tools remain relatively enigmatic, handaxes are often associated with butchery, involving most of the artifact’s use-edge, while cleavers, axes, and adzes are more commonly associated with wood-working, bone-working, and quarrying, employing mostly the distal edge only [44–54]. Performing diverse tasks like slicing, cutting, and scraping with handaxes involves much dexterity, making a CoM closer to the hand advantageous. Meanwhile, the chopping, chiseling, and wedging actions associated with cleavers, axes and adzes are made more effective with their CoM closer to the use edge as more force can be imparted.

An artifact’s CoM can also be used to explore intra-assemblage variability. At Nahal Zihor for example, a Mann–Whitney U test shows that the earlier handaxes from Group A have significantly lower CoM values than those from the more recent Group B (U = 230, d.f. = 88, p<0.001).

Moreover, the degree of dispersion of CoM locations reveals the importance of an artifact’s distribution of mass. An F-test for equality of variances shows that sickle blades have significantly greater variability in the position of the CoM for both the y (F = 16.23, d.f. = 116, p<0.001) and x (F = 5.17, d.f. = 116, p<0.001) axes compared with the spheroids. For sickle blades, which are typically hafted, the distribution of mass is relatively immaterial to the successful use of the artifact. For spheroids, on the other hand, central CoM values likely held functional and/or aesthetic significance.

From these examples alone, it is clear that the location of an artifact’s CoM has substantial technological, functional, and aesthetic significance. For complex, irregular 3D objects made of homogenous material, CoM can only be reliably calculated by analyzing the volumetric distribution of 3D models, and is automatically calculated by *Artifact3-D*.

### 3.3 Manual measurements and Create Cut

**Fig 4:**
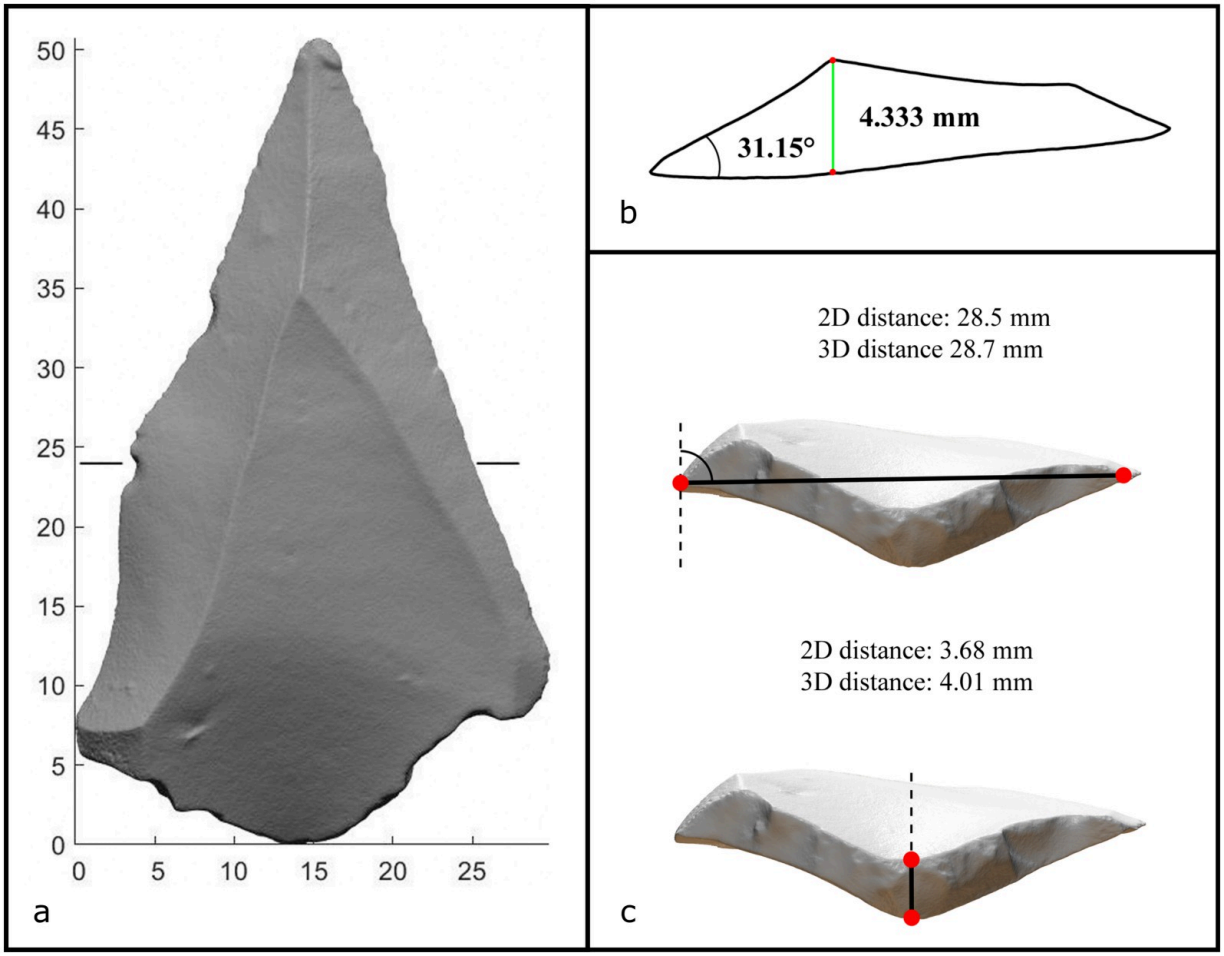
The 3D model of the Levallois flake showing the location of the cut made using the *Create Cut* function (a). The angle and length measurements taken on this cut (b). The platform width and depth distances taken using the *Manual Measurements* function (c).

Aside from this automatic calculation of a suite of metrics, coordinates and indices, a range of miscellaneous values can also be manually calculated (Fig 4). For instance, the *Operation Panel—Manual Measurements* function will return both the 2D and 3D linear distance between any two points on the object, as well as the angle between these two points and the z-axis. The 2D manual measurement computes the 2D linear Euclidean distance on the plane between two points, while the 3D measurement incorporates the z-coordinates of the points also. This function can be used to measure any specific artifact attributes not automatically measured by the software. On flakes, for instance, the platform width and depth (Fig 4d) are commonly desired attributes by lithic analysts that can be used to understand the fracture mechanics of knapping as well as predict the original flake’s mass prior to retouch in order to estimate reduction intensity [55–62]. Of course, the length of any specific attribute of any artifact can be measured using this function.

The *Operation Panel—Create Cut* function will extract a 2D section at a user-defined point along the z-axis. Without 3D analysis methods, section cuts are typically created by archaeological illustrators who must approximate the cut visually, making any subsequent measurements unreliable. Instead, the *Create Cut* function isolates the 3D coordinates of the section cut, providing an accurate and reliable outline of any user-defined section. While this function is useful for documentation purposes, both distance measurements and angle measurements can also be taken on this section (Fig 4b). The 2D coordinates of this cut section can also be exported as a .mat or .txt file for further morphological analysis. The type of cross-sectional morphology has previously been used to quantify the skill of bifacial flaking [63–65] distinguish between different typologies of axes and adzes [46, 66, 67], analyze prismatic blade technology [68–70], and distinguish different types of projectile points [71, 72].

Previously, we used this *Create Cut* function to reconstruct the slab morphology of giant cores at Gesher Benot Ya’aqov [73]. We also used it to measure the edge-angles of side-scrapers and their associated sharpening flakes, thereby reconstructing the skill and intentionality of resharpening practices at Nesher Ramla [74]. For this archaeological research question, 2D angle measurements of accurately rendered section cuts was sufficient to reveal that the inhabitants of Nesher Ramla desired long, straight, and fresh edges. For other research questions, however, a method of angle measurement that incorporates all 3D coordinates is sometimes required.

### 3.4 Angle measurements

Measuring angles between the surfaces of lithic artifacts provides important insights into different aspects of tool manufacturing and use. For example, angle measurements serve as the basis for understanding reduction methods [21, 75–79], tool functionality [51, 52, 80–85], reduction intensity [86–88], how flakes propagate [89–92], and even long-term evolutionary trends in lithic technology [61].

Manually measuring the angles between irregular surfaces of lithic artifacts is often a demanding task and the result is influenced by possible ambiguity in the artifact positioning. Traditional methods of angle measurement have proven to be highly susceptible to systemic, random, and human error [93]. To remedy this, the *Operation Panel—Angle between Surfaces* function of *Artifact3-D* allows the user to calculate the mean angle between two irregular surfaces [21]. By considering the mean angle between two 3D surfaces at multiple distances from the edge, this function identifies the most evenly sloping area on each surface, thereby providing the most reliable estimate of an edge’s angle.

**Fig 5:**
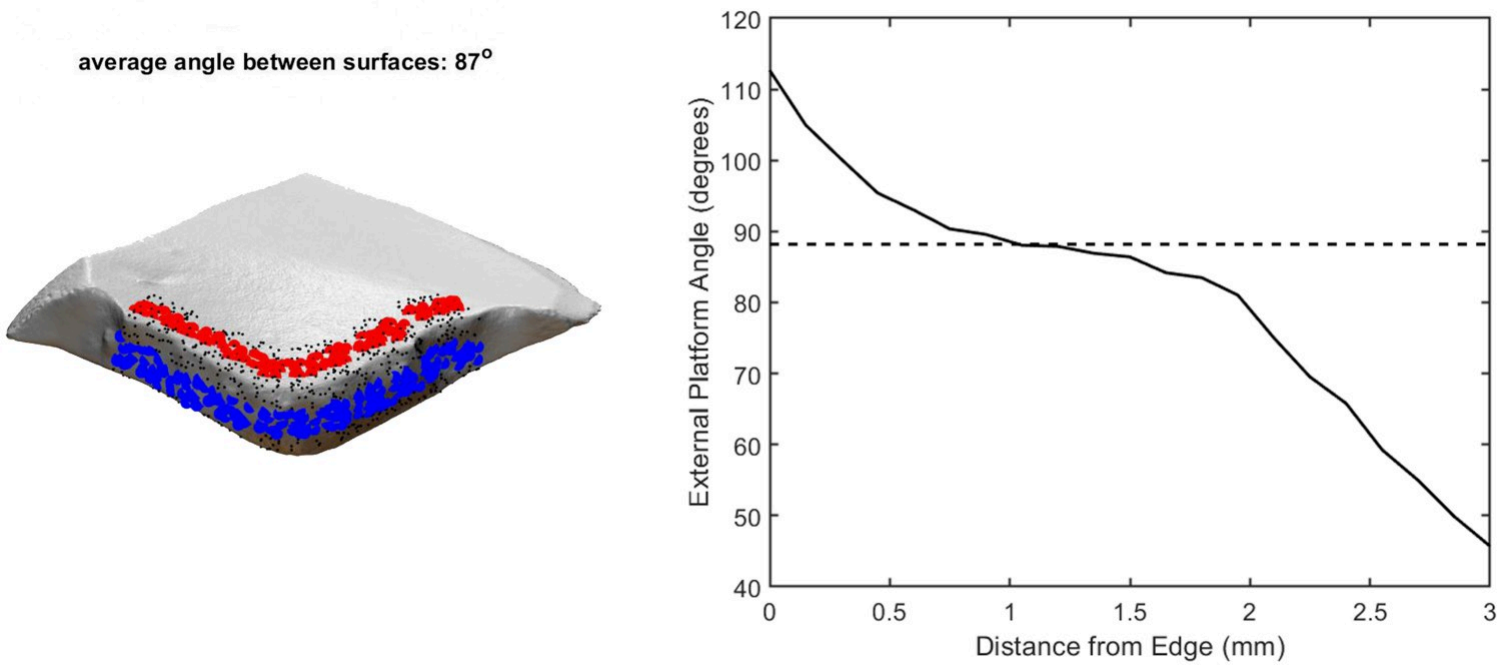
The external platform angle calculation showing the points at which angle is calculated on the platform (blue) and dorsal (red) surface (left), and a plot of the mean angle between the surfaces for different distances from the edge (–), as well as the mean angle returned by the software (—) (right).

The procedure allows users to set the portion of the artifact surface included in the calculation based on two or more points, manually selected along the edge between the surfaces, and a maximum distance from this edge (Fig 5). The angle is then calculated based on the most regular portion of the surfaces at the two sides of the edge. In addition, the function returns the mean angle measured at different distances from the edge, as well as along different consecutive edge segments when the edge is defined by more than two points.

For the example Levallois flake in Fig 5, the edge between the platform and dorsal surface was demarcated, and a maximum distance from the edge of 3mm was chosen. The *Operation Panel—Angle Between Surfaces* function calculates the angle between these two surfaces at multiple points (shown in black) but only returns a mean angle value for the points at a distance from the edge with the least variability. This is to avoid areas of higher variability that may bias the angle result, such as the area closest to the edge, where wear or retouch can alter the angle, or the area farthest from the edge, where negative scar curvature or positive ventral curvature can alter the angle. The plot in Fig 5 demonstrates the extent to which the angle values vary at different distances from the edge. The dashed horizontal line represents the mean angle value at the most evenly sloping area (88.20°).

As an example of the utility of this function, this precise and objective method of angle measurement was applied to the mean edge angle of Epipaleolithic microliths in the southern Levant [21]. Continuity and variability of these edge angle values revealed connections in the manufacturing traditions of different cultural groups, demonstrating the circulation of people and ideas in the region.

### 3.5 Asymmetry

The symmetry, or lack thereof, of archaeological artefacts is a common trait used by archaeologists to infer the manufacturing method, standardization, and even skill of past hominins. Symmetry can serve both aesthetic and functional purposes. The human tendency to aesthetically prefer symmetrical objects has been considered archaeologically, especially pertaining to the symmetry of stone tools [94, 95]. Meanwhile, arrowheads and other projectiles often benefit aerodynamically from symmetry [96] or even strategic asymmetry [97], and marginal benefits in butchery efficiency have been observed for more symmetrical bifaces [98].

In bifaces for instance, asymmetry has previously been calculated by segmenting the artifact longitudinally and comparing the paired segments [50, 99–106], by using the flip test [64, 107, 108], by comparing the volumes of two halves [109], or by segmenting the artifact according to its most symmetrical axis and comparing the asymmetry of the two outline halves [13, 16, 17, 98, 110–112]. The *Artifact3-D* software uses this last method.

*Artifact3-D* isolates a closed curve that defines the outline of the artifact when projected on the ventral plane. Next, a line that separates this outline into halves with the least amount of asymmetry is found. The difference between the two halves of this outline is then quantified according to the formulae outlined by Saragusti et al. [111]. Prior to the proliferation of 3D scanning methods, Saragusti et al. [111, 112] manually extracted 2D outlines of artifacts from plan drawings. We can now automatically project the 3D model onto the x-y plane and extract the precise 2D outline of the artifact.

For bifacial technologies, like handaxes, asymmetry reveals much about the skill and cognition of past knappers. The repeated pattern of bifacial flaking involved in these technologies, designed to achieve a certain shape, means that a measure like asymmetry can allow a rare insight into their intentionality. For this reason, measures of asymmetry are most frequently applied to bifacial technologies, like handaxes and points. To demonstrate the broader utility of this function, we instead apply this metric to Levallois flakes to test whether asymmetry can be used to distinguish the skill level of knappers based on their flakes alone.

We compared Levallois flakes produced by two experimental knappers of different skill levels to explore how skill influences the asymmetry of recurrent Levallois flakes. For the experiment, highly cryptocrystalline flint from the same source was knapped using the same quartzite hammerstones. The more experienced knapper has more than ten years of knapping experience, while the less experienced knapper has only three. Both produced 37 Levallois flakes using a recurrent centripetal method on several cores. Following archaeological and experimental reconstructions of Levallois reduction sequences [113–118], all cores were exploited until no more recurrent Levallois flakes could be produced.

**Fig 6:**
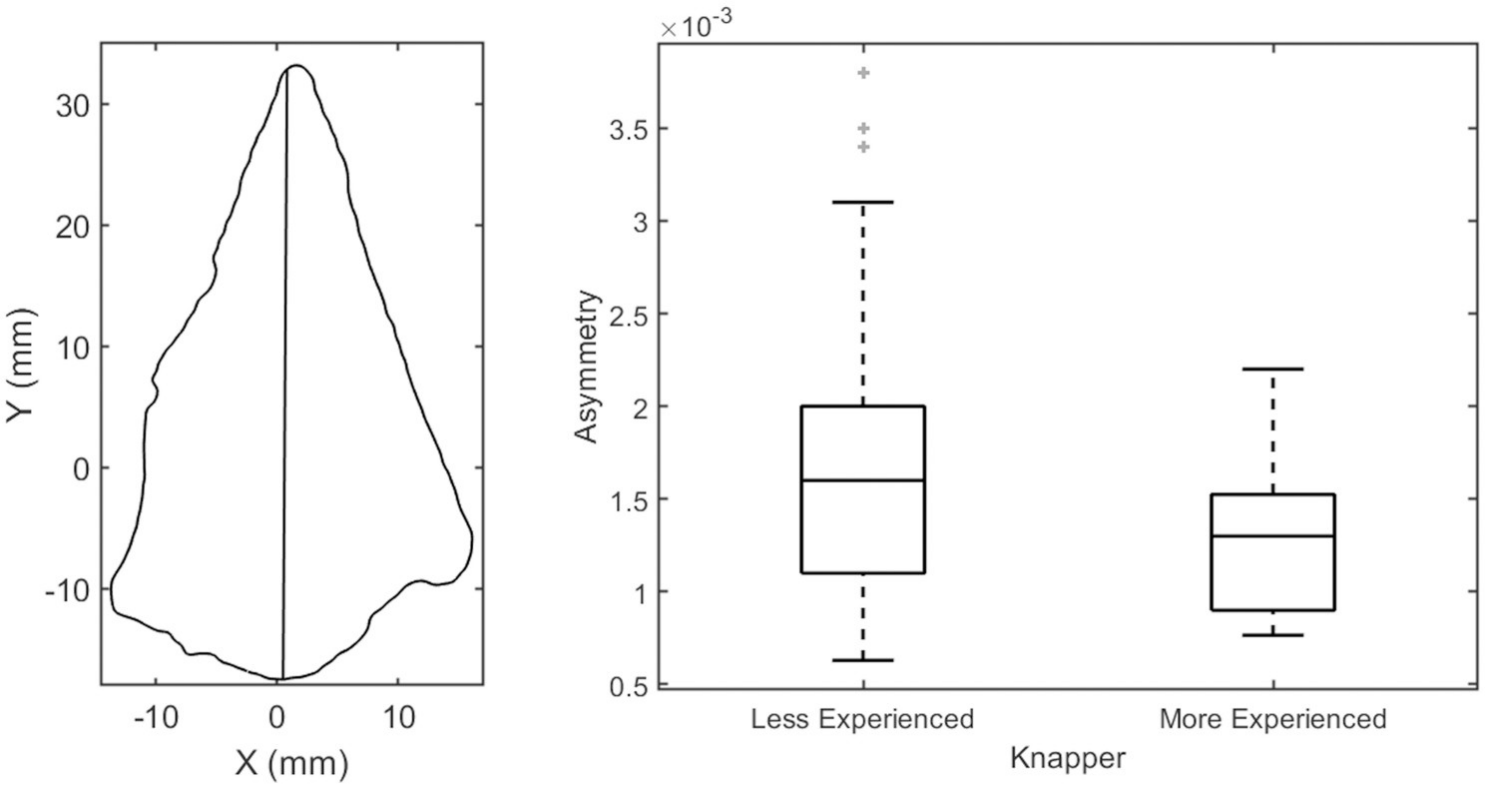
Plan view of the example Levallois flake with the line that separates the artifact outline into halves with the least amount of asymmetry (left), and a boxplot of the asymmetry values for the less and more experienced knappers (right).

Fig 6 shows the axes of symmetry for the example Levallois flake, demonstrating how the two halves of the outline are segmented for comparison. The boxplot in Fig 6 shows that the recurrent Levallois flakes produced by the more experienced knapper were significantly less asymmetrical compared with those produced by the less experienced knapper (U = 451, d.f. = 73, p = 0.011). Previously Eren et al. [119], found that the asymmetry of Levallois flakes is influenced by knapper skill level, but statistical comparisons were inconclusive. Here, we confirm their expectations that asymmetry is lower with a higher skill level.

Of course, this measure of asymmetry can be applied to any lithic or non-lithic artifacts. For instance, we previously used this measure to explore the role of post-depositional damage to handaxe outlines [17], as well as chronologically differentiate two different handaxe assemblages from Nahal Zihor [16]. Although most lithic analyses that incorporate asymmetry are conducted on complete bifacial tools, here, we demonstrated the utility of this asymmetry metric for flakes. This opens new avenues for exploring skill level in the past as flakes are overwhelmingly more abundant in the archaeological record.

### 3.6 Scar identification and analysis

*Artifact3-D* also provides a function for the automatic segmentation of scars or ridges on lithic artifacts. These ridges exist on core surfaces, on the dorsal surface of flakes, and on the retouched edges of tools. In each instance, much technological information can be derived from the position, number and size of these scars, such as reduction sequences, reduction intensity and knapping strategy. For instance, analysis of scar abundance on 3D scans has previously been used to calculate reduction intensity [120–124], while scar directionality has been used to infer reduction strategies of cores [125, 126]. Surface area has also proven particularly useful in improving our ability to calculate the reduction intensity of cores [120, 127, 128], core-tools [122, 124, 129, 130], and flake-tools [86, 87, 92, 131].

This scar segmentation function (*Scars Panel—Analyze*) estimates the ridges between the scars as continuous areas of high local curvature. Local curvature is a measure of how much a surface deviates from a flat plane in a small neighborhood surrounding a point. For differentiating between ridges and less informative surface anomalies, this function seeks continuities in this local curvature. Importantly, the thresholds for defining what is sufficiently high curvature to warrant adding a ridge are customizable (*Scars Panel—Tune Params*).

Then, the function searches for an optimal division of the surface into continuous parts (scars) which cross the ridges as little as possible. This stage involves 3D mesh algorithms such as geodesic distance for dividing the surface wisely, and optimization algorithms such as alpha-expansion for optimizing the final result. Based on the boundaries between the optimized scars from the previous step, lines are thereby overlain on the scanned artifact to represent the ridges left behind during lithic reduction (for technical details see [18, 19]). As different raw materials behave differently, and areas of concretion or wear can obscure existing scars, the ridges can be merged or removed manually.

Beyond simply identifying scars and computing metrics from them collectively, high-resolution data can also be extracted from each scar. *Artifact3-D* exports specific mathematical properties of the two largest scars, including 3D surface area, boundary attributes, maximal curvature, and compactness. Maximal curvature is a measure of how much a point and its nearest neighbors deviate from a flat plane. *Artifact3-D* outputs the mean, median, and standard deviation these maximal curvature values for all points on a surface. Compactness is the ratio of an object’s area to the area of a circle with the object’s perimeter. It is a measure of the uniformity of an object’s outline. A circle possesses a compactness value of 1.

**Fig 7:**
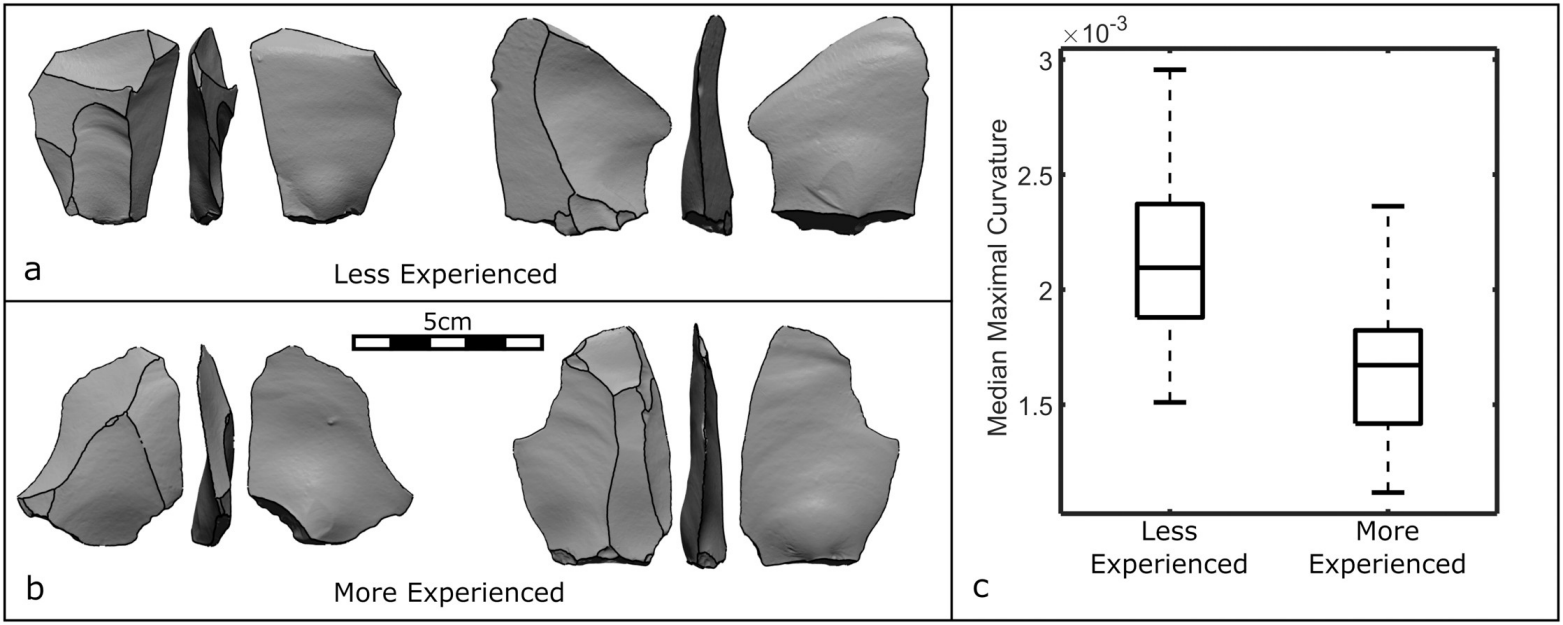
Levallois flakes from the less experienced (a) and more experienced (b) experimental knappers, showing the result of the scar segmentation function, as well as a boxplot of the median maximal curvature values for both knappers (c).

To demonstrate the power of these attributes for addressing archaeological questions, we compared the recurrent Levallois flakes (Fig 7) produced by the two experimental knappers discussed above (section 3.5). We seek to distinguish these two samples of flakes based on the subtle attribute of median maximal curvature alone.

The flakes made by the more experienced knapper possessed less curvature on their ventral surfaces (Fig 7c), with significantly lower median maximal curvature values (U = 217, d.f. = 73, p < 0.001). Less experienced knappers are more likely to impart too little or too much force into the core resulting in aberrant (hinge or step) terminations and pronounced ripples that deform ventral surfaces and alter the amount of curvature.

Previously, knapping skill has most often been quantified by the frequency and severity of knapping mistakes and inefficiencies [65, 119, 132–138]. Here, we demonstrate how a precise mathematical feature of ventral surface morphology alone can distinguish knappers of different skill levels.

**Fig 8:**
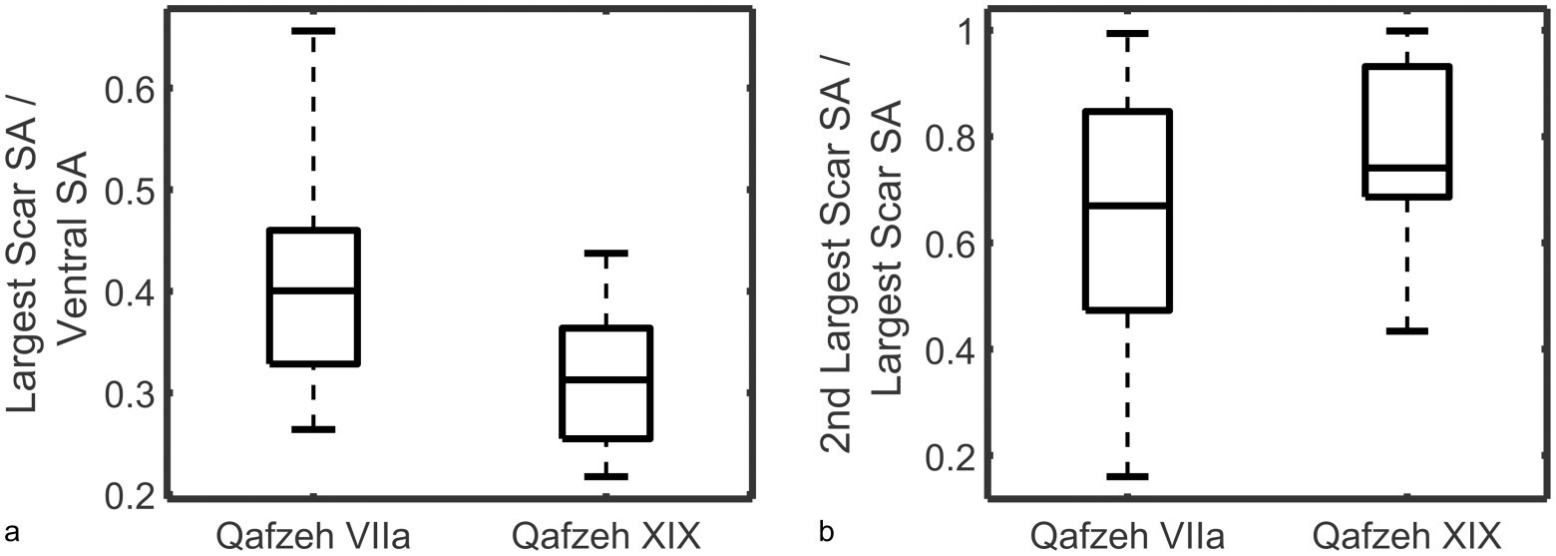
Boxplots for layers VIIa and XIX of Qafzeh Cave, showing the ratio in surface area between the largest dorsal scar and ventral surface (a), and the ratio in surface area between the second largest and largest scar (b).

Alongside values like curvature, *Artifact3-D* also calculates the surface areas of all the surfaces demarcated by the scar ridges. Precise surface area measurements can aid in size comparisons, allometric calculations, and stone tool reduction intensity metrics. On flakes, surface area comparisons can also reveal much about the scar patterning and reduction sequence of a technology. To demonstrate this, we compared two samples of unretouched Levallois flakes from an earlier (XIX) and later (VIIa) layer of Qafzeh Cave, revealing marked differences in scar patterns (Fig 8).

The surface area of the largest dorsal scar in layer XIX was consistently close to a third of the ventral surface area, while in layer VIIa this ratio was significantly higher (U = 135, d.f. = 51, p < 0.001). Meanwhile, the surface area ratios between the second largest dorsal scars to largest dorsal scars in layer XIX were significantly higher than those in layer VIIa (U = 208, d.f. = 51, p < 0.05). This means that the Levallois flakes in layer VIIa were more often comprised of one large flake dominating the dorsal surface. In comparison, the Levallois flakes of layer XIX possessed much more evenly sized scars, and therefore a more invasive reduction sequence.

These differences reflect the effectiveness and invasiveness of the knapping strategy employed in the different levels. The flakes removed from a Levallois core’s upper surface are designed to add curvature to the core and help predetermine the next recurrent Levallois flake removal. Ideally, these removals intersect with the previous recurrent Levallois removal resulting in relatively regular dorsal scar patterns. Archetypal depictions of Levallois flakes tend to show dorsal surfaces comprised of approximately three similarly sized scars. In contrast, the large negative scar that dominates the Levallois dorsal surfaces of layer VIIa can often be explained by a less invasive flaking strategy, whereby a larger portion of the previous recurrent Levallois scar remains on the dorsal surface of the next recurrent removal. As such, these surface area ratios reveal much about the invasiveness and patterning of flake scars at Qafzeh.

The nature of the lithic variability in the different layers of Qafzeh has attracted much discussion. It has been suggested that the variability between the lower and upper levels can be explained by the presence of different cultural entities [139], or different seasonal patterns [140]. A comprehensive analysis of the Qafzeh lithic assemblage revealed that much complex variability exists among the different levels and that simple dichotomies are likely insufficient to explain the variability throughout the sequence [141, 142]. Precisely quantifying the invasiveness of flake scars using *Artifact3-D* can potentially add to this understanding of the flaking strategies employed by the Qafzeh hominins and help quantify the technological organization at the site.

Here we have demonstrated how even the most subtle differences in ventral and dorsal surfaces can be used to distinguish Levallois flakes from different archaeological layers, and even distinguish between knappers of different skill levels. Less experienced knappers are more likely to produce ventral surfaces with large bulbs of percussion and aberrant terminations, making their curvature values distinct from flakes produced by more experienced knappers. Meanwhile, the dorsal surfaces of Levallois flakes from Qafzeh layer XIX involved a more formalized and evenly invasive system of preparatory flake removals than the more recent Levallois flakes from layer VIIa.

## 4. Illustration and documentation

The *Artifact3-D* software also provides automated functions for visualization and documentation of artifacts. Most of the analysis functions outlined above also have documentation components. The orthogonal measurements, CoM, edge angle calculation, and automatic scar segmentation are all represented on the interface and can be exported for illustrative purposes. For example, the scar segmentation function has obvious implications for automating and making objective the process of lithic illustration, which primarily relies on the demarcation of scar boundaries.

This procedure in illustrating the potential for 3D scans helps begin replacing traditional drawings and schemas [143]. While we do not yet recommend discarding pen and drafting paper altogether, we hope to offer another step in the direction of automating and removing subjectivity from artifact visualization.

*Artifact3-D* offers users the ability to create and edit plates comprised of multiple artifacts for publication *Plate Panel*. While these functions automatically create various illustrations, they are also completely customizable using *Plate Panel—Drawing Tools*. The positioning of artifacts can be altered as desired, and lines, arrows or coloring can be included to represent features like breakages, cortex, gloss or polish.

## 5. Discussion and conclusions

The *Artifact3-D* software provides a suite of analysis and documentation procedures that can accommodate a range of research questions for lithic analysis, and beyond. The case studies explored above are by no means an exhaustive list of the possible applications of the *Artifact3-D* software, but rather serve as examples of how the software can be employed to precisely quantify variability among different samples and address both experimental and archaeological research questions.

For instance, we explored how CoM values can be used to investigate the functionality of different types of bifaces, and even distinguished between earlier and later handaxes at a single site. The CoM of artifacts was also used to understand how the distribution of mass was significant to the typology and function of spheroids, but insignificant for sickle blades. Moreover, we showed how manual measurements of length, surface area and the section cut can be used to explore elements of technological organization like reduction sequences and reduction intensity. The precise and objective edge angle measurement procedure was also used to track the manufacturing traditions of different Epipaleolithic cultural groups in the southern Levant. Meanwhile, asymmetry was used to differentiate Levallois flakes made by experimental knappers of different skill levels. Lastly, the scar segmentation procedure was used to further distinguish the Levallois flakes made by the less and more experienced knappers, while also distinguishing the Levallois flakes from different layers of Qafzeh Cave.

Here we have shown the utility of the *Artifact3-D* software in providing precise and repeatable measurements, including orthogonal distances, surface area, volume, CoM, edge angles, asymmetry, and scar attributes. As examples, we have applied these measures to Levallois flakes, Acheulean bifaces, Neolithic bifaces, spheroids, sickle blades, but they could be applied to any lithic or non-lithic artifact. Altogether, these case studies demonstrate how the *Artifact3-D* software can be used to address archaeological research questions by quantitatively measuring variability within and between artifact assemblages.

Finding a shared scheme for describing variability remains one of the main problems in artifact analysis. For many artifact types, there remains confusion surrounding typologies and nomenclature, and we lack a standard quantitative definition of artifact "variability". The case studies above showed that *Artifact3-D* can help construct a more quantitative approach for presenting the degree of “variability” between artifacts and assemblages.

The *Artifact3-D* toolkit encompasses the entire workflow of artifact processing, analysis, documentation, and publication. Once users acquire 3D scans, *Artifact3-D* processes these 3D models, positions them consistently based on their intrinsic geometric properties, and generates views, measurements and sections that have been selected algorithmically, without user input, bias, or interpretation. This unbiased system of object manipulation and orientation forms the basis for a series of subsequent measurements and analyses that can quantify any aspect of the artifact’s morphology.

Whereas traditional artifact analysis involves an unavoidable and significant degree of inter-observer error, this software completely removes any measurement errors introduced by different analysts or subjective parameters. Attempts at quantifying the extent of inter-observer variability in archaeological analysis have shown that traditional methods of measurement can introduce significant random and non-systematic errors that influence analyses and interpretations [60, 93, 144–147]. Even a simple length measurement of an artifact taken with calipers can vary significantly based on how the analyst orients the object and where they choose to position the two jaws of the calipers. More complex and subjective measurements, like edge angles involve even greater levels of inter-user variability, with up to 18.8 deg^2^ of intra-observer variance [21, 93].

With *Artifact3-D*, these random and non-systematic errors are entirely avoided as the system of object orientation and measurement can be automated based on intrinsic geometric properties of the object. Additionally, several features are available for users to analyze artifacts based on global and local shape parameters. Regardless of who is using the software, if they employ the same procedure, *Artifact3-D* will return the same results for the same object every time.

Importantly also, this automatic and standardizable workflow can be scaled-up to accommodate very large sample-sizes and generate large amounts of data. Despite the influx of big-data methods in other fields of science (e.g. [148, 149]), their proliferation has been slow within archaeology owing to the fragmentary and varied nature of the archaeological record. While there have been some interesting recent attempts at applying machine learning to the study of lithics [150–152], hominins [153–155], fauna [156], pottery [157], art [158, 159], built structures [160], geoarchaeology [161–164], and remote sensing [165], our sample sizes and training sets are often too small for widespread use of these methods. Moreover, many of these studies require drastic reductions in resolution while also remaining susceptible to variability in orientation and inter-user variability.

To counter these problems, we require means of obtaining large and standardized data sets. *Artifact3-D* contributes to this process by providing a suite of fast, standardized, repeatable, and precise analyses that can be used to begin generating the type of large databases that lend themselves to big-data methods like data mining, cluster analyses, predictive modelling, and machine learning.

Fortunately, 3D scanning is fast becoming widespread in archaeology. As entry-level scanners are becoming more affordable, this method is increasingly becoming a cost-effective alternative to existing documentation and analysis methods. Already, we have distributed *Artifact3-D* to many archaeological centers worldwide who have begun incorporating it into their workflows (e.g., [73, 166–170]), and we hope that it will become a common tool for others. The natural next step is to design a web-based archaeological database that integrates the features of *Artifact3-D* for the documentation and analysis of the web-datasets. It should comply with industry connectivity standards in such a way that querying it and displaying the results can be programmatically integrated within this web interface.

*Artifact3-D* represents an important step in merging archaeology with the computational sciences. We hope to see computational archaeology become the standard practice for archaeologists in both the field and lab. Computational archaeology can revolutionize traditional archaeological practices, and is increasingly becoming involved in the excavation, documentation, analysis, database creation, and publication of research results. *Artifact3-D* provides solutions at all stages of this workflow and can be used to quantify artifact variability objectively and precisely, as well as address novel archaeological research questions that have otherwise reached an impasse.

## Supporting information

S1 File *Artifact3-D* user manual (future updated versions at https://sourceforge.net/projects/artifact3-d/). (DOCX)

S2 File A 3-D scan used for illustrating the various functions of the software. (WRL)
