# Supplementary material for: Artifact3-D: New software for accurate, objective and efficient 3D analysis and documentation of archaeological artifacts
Source: PLoS One. 2022 Jun 16;17(6):e0268401. doi: 10.1371/journal.pone.0268401 (PMC9202890; doi:10.1371/journal.pone.0268401)
Supplement: S1 File — (DOCX) [file pone.0268401.s001.docx]

***Artifact3-D* User Manual**

**Overview**

The *Artifact3-D* software was developed at the *Computational Archaeology Laboratory* of the *Institute of Archaeology*, *Hebrew University of Jerusalem*. The software provides analysis functions that are repeatable, objective and precise, but also customizable. Functions are also available for the neat documentation of artifacts. Depending on your needs, individual artifacts can be imported, analyzed and their documentation exported, or entire assemblages can be automatically analyzed. *Artifact3-D* is not a commercial product. It was designed to fulfil archaeological research questions and has evolved over the last decade. It is free for personal academic use.

**Importing Scans**

The software supports 3D models in .wrl, .ply, or .stl formats. Other formats can be converted to these supported formats using your scanner’s native software or third-party software like *MeshLab*. Within the *File Panel* on the left side of the interface, there are three options for importing 3D scans for use within *Artifact3-D*. For importing new artifacts one at a time, use the *Create Qins File* button, and if returning to a previously loaded artifact, use the *Load Object* button. For automatically importing, analyzing and exporting multiple artifacts at a time, use the *Process Directory* button.

Create Qins File

Once you have a .wrl, .ply, or .stl file you can import it individually into Artifact3-D using *Create Qins File*. In the file browser window that opens you can use the drop-down menu to select the file type of your 3D model. Once you have selected and opened your model, the software will ask if you want to close the model. For most artifact types, it is best for the software to close the model to ensure the measurement and center of mass calculations are as accurate as possible. Once imported, the software will automatically save a Qins file (.mat) in the same folder as your original scan.

Load Object

If you are returning to a model you previously imported and for which you already have a Qins file, you can use the *Load Object* button to more quickly access your model. This function will also import any previous changes in orientation or processing you made.

Process Directory

The *Process Directory* button allows you to import multiple files at once. It can also automatically create a section cut at one fifth, four fifths, or the center of mass of your artifact. This function will also automatically generate the output from the *Process Object* function (described below), including images of each model in all orthographic projections as well as images and data files of the automatic measurements. Once clicking the *Process Directory* button, simply select the folder containing your models and follow the prompts. Depending on the number and size of your models, this process could take some time, but a progress bar will keep you updated with its status. In the folder that originally contained your models, you will find a folder of your *Original Data Files* and a folder of any models that were *Not Processed*. The *Not Processed* folder will also contain text files containing the error messages relating to each non-processed object. See the troubleshooting section at the end of this document for common workarounds for these errors.

Analyze Directory

The *Analyze Directory* button allows you to conduct the scar analysis function on a directory of already created .qins files. Simply select the folder containing your .qins files and ScarsQins files will be generated. The next time you load your object, the scar segmentation will be loaded also. See the Automatic Scar Segmentation section below for details on how to modify the scars and export data relating to the scars.

**Positioning**

Now that your model is imported into the software you should see it in two plan views, two section views, and a profile view, as well as a section cut view if you selected this option using the *Process Directory* function. The software will have automatically positioned your artifact using one of the automatic positioning functions described below (*Normal Positioning*). These automatic positioning functions allow for objective and repeatable artifact analysis and can be found in the *Positioning Panel*. These automatic positioning functions were designed for artifacts with lateral symmetry, so may not be ideal for all artifact types. The *Normal Positioning* and *Inertia Positioning* buttons perform two different methods of automatic positioning (see the main text for the methodological differences between these orientations). If you wish to change your artifact’s orientation, the *Manual Positioning* function in the *Operation Panel* allows for coarse and fine orientational changes.

Manual Positioning

If none of these automatic positionings are suitable, then you can use the *Manual Positioning* function. Within the new window that opens, the *Free Rotate* button allows coarse rotation around any axis. Meanwhile, the red arrows allow the object to be rotated around the x-, y- and z-axes in increments of either 1, 2, 5, 10, 45 or 90 degrees, depending on your selection in the *Rotations Steps (degrees)* dropdown box. Once satisfied with the position, clicking the *Save Current Positioning* button followed by *Quit*, will return you to the primary interface with your model’s new orientation.

**Analysis**

Once your artifact has been imported and suitably oriented, you can conduct a number of analyses within the *Operational Panel* and *Scars Panel*.

Process Object

Within the *Operational Panel*, the *Process Object* function undertakes a series of automatic measurements and analyses. Once you press *Process Object* you will be given a choice between the *boundary* or *old contour* method. We recommend the newer *boundary* method. The *old contour* method is an older version, kept here in the interests of backwards compatibility for those already using the program. The *Process Object* function provides measurements (mm) of the artifact’s maximum length, width and thickness, as well as its width and thickness at its one-fifth, four-fifths, and midsection. Measurements are also recorded relating to the object’s volume, center of mass and virtual bounding box. This function outputs two images; one of the artifact in all orthogonal projections and the other containing a representation of all automatic measurements. These are output in both .jpg and .fig formats. The automatic measurements can also be saved to an excel file at the prompt. Lastly, the object’s contour data can also be saved, providing a .mat file of the object’s outline in 2D coordinates for both plan and profile views. All output files are saved into the same folder as the original .qins file.

Manual Measurements

The *Manual Measurements* function allows additional linear measurements to be taken on your artifact. Ensure the feature you wish to measure is visible on the primary view of your object in the main window. If not, use the *Manual Positioning* button to rotate your object, ensuring you save this orientation before exiting to the main window. Once the orientation is suitable, the *Manual Measurements* button will open a new window in which two points on your artifact can be selected by following the prompts. The software will provide the distance (mm) and angle (degrees) in relation to the z-axis between these two points in both 2D and 3D space. These values can be exported to a text file, and the resultant image saved in .tif format using the buttons on the bottom right of the window. Closing the *Manual Measurements* window will return you to the primary interface.

Create Cut

The *Process Directory* function can create a cut in section view at one fifth, four fifths, or the center of mass. The *Create Cut* function allows you to create this section cut at any other point along the z-axis. You will be prompted to choose between a section cut that is looking up or down the z-axis. The software will then produce a window containing your object in all orthogonal projections, and a crosshair cursor will allow you to select whichever point on whichever projection at which you wish to create a cut. For reference, on the two plan views, red lines will indicate maximum length and width, while green lines represent the width at one-fifth, four-fifths, and midsection.

Once you select and confirm a point at which to create a cut, you will be returned to the primary interface and asked if you want to manually measure the resultant cut. If you select *yes*, a new window will appear in which you can measure distance by selecting two points on the 2D representation of the cut, or measure an angle by selecting three points. The resultant figure can then be saved in .tif format. You will also be prompted as to whether you want to save the 2D coordinates of this contour in .txt or .mat format. Once you close this window, or if you selected *no* when asked if you want to manually measure the cut, you will be returned to the primary interface, where the cut has been added to the orthogonal views. Please note that if you want the section cut to be included in the automatic analyses of the *Process Object* function, ensure you conduct the *Create Cut* function first.

Calculate Angle Between Surfaces

This function allows you to calculate the mean angle between two irregular surfaces, based on the most regular portion of these two surfaces. In the user-interface window that appears you can position the object so that the two surfaces you want to calculate an angle between are most visible by selecting the *Rotate 3D* icon (located in the window frame near the upper-left corner). To measure the angle, select the *Calculate angle with h_test* button. This function is the most up-to-date procedure with the most features. The *Calculate angle* and *Calculate multiple angles* buttons are legacy functions.

The *Calculate angle with h_test* procedure requires the user to insert a series of parameters in the dialog box that appears. The *max h1* value represents the maximum distance (in mm) from the edge of the calculation area. The ‘dilute factor’ is used to calculate the fraction of the vertices in the area used for calculation. The optimal dilute factor value will automatically appear in the dialog box but can be changed if desired.

The edge between the two surfaces is defined by two or more points, that can be

either selected manually or loaded from a previous measurement. To select points manually, enter the number of points that you will use to define the edge (minimum 2) and click *OK*. Then, select the points along the edge with the crosshair cursor (left click). If happy with your selection, confirm with *Yes*, or you can reapply the points by clicking *No.* If you have previously performed an angle measurement on the current object and saved the points as a set of .mat variables, it is possible to load the points from the previous measurement. Within the file dialog box, find and select the .mat file from which you want to load the points. The points used in the previous measurement will appear along the edge. If happy with these points, confirm with *Yes*.

Having selected your points that define the edge of the two surfaces between which you wish to calculate the angle, you can choose between the *Mean angle between surfaces* or the *Angle between mean surfaces* procedure. The first, calculates the mean angle between the normals of the two surfaces, while the latter calculates the angle between the mean normals of the two surfaces. For most objects, the results will be generally similar.

After selecting your method, the angle will be calculated, and the result can be saved as a .mat file, either as a new file or added to an already created set of angle measurements, or it can be saved as an .xlsx file. The .xlsx file contains the mean angle measurement, and its standard deviation, of each consecutive segment of the edge, as well as the length and xyz coordinates of each segment.

Asymmetry

The *Asymmetry* function will find the best line of symmetry for the primary orthogonal projection of your artifact, and calculate the amount of asymmetry between the portions of the 2D contour on either side of this line. First, you will be asked to enter a smoothness parameter. This is the number of Fourier coefficients used to reconstruct the 2D contour. Lower values will result in a smoother contour, while higher values will better resemble the full variability of your actual contour. Once selected, the program will generate a plot of your object’s contour as well as the line of most symmetry. The program will also report the amount of asymmetry on either side of this line.

Center of Mass

The center of mass of your object, calculated using the object’s volumetric distribution, can be computed and represented by a red dot on your artifact using the *Mark Centre of Mass* button in the *Viewport Panel*. Please note that the coordinates of the center of mass of your artifact will always be 0, 0, 0. Information relating to the center of mass’s relationship to the bounding box can be found in the exported material resulting from the *Process Object* or *Process Directory* functions.

Automatic Scar Segmentation

To overlay automatically generated scar boundaries on your model, select the *Analyze* button in the *Scars Panel*. This function may take several minutes depending on your model size. Once finished, the software’s attempt at identifying scar boundaries will be displayed. If scar boundaries have been drawn where there is no scar, or if a scar has been ignored, the *Merge* and *Tune Params* functions can refine the scar boundary selection.

The *Tune Params* function allows you to change the curvature thresholds used to demarcate scars using four horizontal scroll bars. A plot of your object is shown on the right, as a guide for how changing these thresholds will affect the final scars. Once you have selected your new thresholds, click *Analyze* and the scars will be recalculated.

The *Merge* function opens a new window in which the object can be rotated to your desired view using the *Rotate 3D* icon. Then, you can select two or more scars using the *Data Tips* icon. Both the *Rotate 3D* and *Data Tips* buttons are located in the top right of the *Merge* window. If these options are not visible, hover your cursor over your artifact and several icons should appear in the top right (*Save*, *Brush Data*, *Data Tips*, *Rotate 3D*, *Pan*, *Zoom*, and *Restore View*). Use the Data Tips cursor to select an area of your object within one of the scars you wish to merge. To select the other scars, you wish to merge with this first scar, hold the *alt* key while left-clicking to select more than one scar area. Once you have selected the areas you wish to merge, click the *Merge* icon at the top left of the window (pink icon). To recombine previously merged scars, you can use the *Data Tips* to select the areas you wish to unmerge, then select the *Unmerge* icon (diamond). You can also delete all merging information and return to the original scar boundaries generated by the *Analyze* function by selecting the *Delete* icon (cross).

**Documentation**

Creating Plates

As well as providing automatic and customizable analysis functions, *Artifact3-D* supports the creation of plates for the neat presentation of multiple artifacts for publication. Among the buttons in the *Plate Panel*, *Create New* allows you to open a blank plate to which you can add multiple artifacts. Previously created plates can be imported using the *Load Plate* function, allowing you to add to or edit your existing plate. In the *Plate Panel*, *Add Current View to Plate* will place your currently loaded artifact into your new plate. As you load new objects using the *Create Qins File* or *Load Object* functions, your existing plate will remain so you can add new artifacts. Artifacts can be added or removed, the order of artifacts changed, and the plate saved using the *Plate Panel*.

Additional plate editing functions can be found by pressing the *Plate Drawing Tools* button. In the window that appears you will be able to *Add an Arrow*, *Add a Line*, and *Add Surface Area* to represent breaks, impact fractures, cortex, gloss and polish etc. Simply follow the prompts in the upper left dialog window. The appearance of dots, lines and surfaces can also be edited. In the current version of the software, these lines and surfaces can be added as black features only. Once complete, you can *Save Final Plate* in .tif format, and return to the primary interface.

Crop

You can use the *Crop* function if you wish to view your model after a portion of it has been cropped or sliced away. This is not to be confused with crop functions that remove extraneous portions of your model or portions of background that were scanned. You can do so in your scanner’s native software or a third-party software like *MeshLab*. Once you select the *Crop* button, a new window will open, and you can use the *Rotate* button to rotate the model and identify the portion of model you wish to remove. Next, select *Create Cut Line* and follow the prompts to select two points along which a line will be created, then select the portion of the model you wish to discard using the crosshair cursor. You can then orient the remaining model as you wish, using the *Rotate* and *Cut Normal Views* buttons, and export a .tif image of the cropped model. This cropping will not affect the model on the primary interface.

**Trouble Shooting Common Errors**

When importing 3D scans, you may encounter the following error messages

- “EIG must not contain NaN or Inf”
- “Index exceeds matrix dimensions”
- “Subscript indices must either be real positive integers or logicals”

These errors are mostly caused by poor quality scans or scans that need pre-processing and cleaning before importing to *Artifact3-D*. The following are some simple workarounds that will usually fix these issues. First, ensure your file is in the correct .wrl, .ply, or .stl format. Next, some scanners will create 3D models with unnecessarily large file sizes. To make scans more manageable in *Artifact3-D* you may wish to reduce the file size (e.g. in MeshLab: *Filters* > *Remeshing, Simplification and Reconstruction* > *Simplification: Quadratic Edge Collapse Decimation*). Please also ensure your model is closed and does not contain any non-manifold edges or extraneous portions of your scan (e.g. the background). Programs like *MeshLab* have functions for closing holes and merging any non-manifold edges etc.

The .ply format will allow you to analyze and document color scans. If importing .ply scans without color data, please ensure that you deselect the color option when exporting your model from your scanner’s native software or a program like *MeshLab*. Otherwise the lighting of the model will not appear as desired. Generally, we recommend using .wrl or .stl files for non-color models.

Lastly, some functions of this program may take time to run, depending on your computer and the file size of the scans. Where possible, functions will update you on their status with a progress bar. However, once you have begun a function, please give it time to work. Clicking on other functions while one is working may cause the software to freeze. If stuck or frozen, simply exit and restart the program.
